# Human adipose stromal-vascular fraction self-organizes to form vascularized adipose tissue in 3D cultures

**DOI:** 10.1038/s41598-019-43624-6

**Published:** 2019-05-10

**Authors:** Sandra Muller, Isabelle Ader, Justine Creff, Hélène Leménager, Pauline Achard, Louis Casteilla, Luc Sensebé, Audrey Carrière, Frédéric Deschaseaux

**Affiliations:** 1 STROMALab, Etablissement Français du Sang-Occitanie (EFS), Inserm 1031, University of Toulouse, National Veterinary School of Toulouse (ENVT), ERL5311 CNRS, Toulouse, France; 2 LBCMCP, Centre de Biologie Intégrative (CBI) CNRS, University of Toulouse, Toulouse, France; 3 LAAS-CNRS University of Toulouse CNRS, Toulouse, France

**Keywords:** Stem cells, Cell biology

## Abstract

Native human subcutaneous adipose tissue (AT) is well organized into unilocular adipocytes interspersed within dense vascularization. This structure is completely lost under standard culture conditions and may impair the comparison with native tissue. Here, we developed a 3-D model of human white AT reminiscent of the cellular architecture found *in vivo*. Starting with adipose progenitors derived from the stromal-vascular fraction of human subcutaneous white AT, we generated spheroids in which endogenous endothelial cells self-assembled to form highly organized endothelial networks among stromal cells. Using an optimized adipogenic differentiation medium to preserve endothelial cells, we obtained densely vascularized spheroids containing mature adipocytes with unilocular lipid vacuoles. *In vivo* study showed that when differentiated spheroids were transplanted in immune-deficient mice, endothelial cells within the spheroids connected to the recipient circulatory system, forming chimeric vessels. In addition, adipocytes of human origin were still observed in transplanted mice. We therefore have developed an *in vitro* model of vascularized human AT-like organoids that constitute an excellent tool and model for any study of human AT.

## Introduction

3-D type cultures have sparked great interest in basic research labs and for translational science. This interest is probably due to the limitations of 2-D cultures with conditions very different from native (*in vivo*) tissues, which may lead to untransferable results to *in vivo* situations^1^. Regarding native tissues, all cells reside within a 3-D complex environment composed of a large number of extracellular matrix molecules and are dispersed geometrically in 3-D space in the correct place. This situation offers biophysical interactions that induce specific biological responses^2^. Overall, these structures build up to give rise to functional organs.

2-D culture systems provide relevant models for research studies but are too simplistic and do not mimic the composition and spatial structures found *in vivo*. Therefore, labs have begun to develop 3-D culture types for many epithelial and non-epithelial tissues to resume the native tissue function, its cellular heterogeneity as well as the formation of specific niches where 3-D architectures are crucial^1,3,4^. Adipose tissue (AT) has a crucial role in the maintenance of whole body metabolic homeostasis^5^ and attempts to culture adipocytes in 3-D have been described^6–9^.

Human white AT is highly vascularized, and its vasculature is essential for functioning. Indeed^10^, each adipocyte is in close contact with nearby endothelial cells (ECs). This contact allows for the blood flow to fetch nutrients, oxygen, growth factors, hormones, cytokines (e.g., adipokines) and other cells (e.g., immune cells) required for AT homeostasis. ECs also constitute a niche for adipocyte progenitors (APs) because of their perivascular/endothelial origins^11–14^. The crucial role of vascularization is also true for the biology of beige APs^15^ and for transdifferentiation of some “white” adipocytes into beige adipocytes. These latter express the uncoupling protein-1 (UCP1) following noradrenergic stress, and assume a role in non-shivering thermogenesis, as classical brown adipocytes present in brown AT^5,16,17^. In addition, direct observations in human samples or the use of lineage-tracing technologies in mouse models showed that during development and AT expansion in adults, angiogenesis and adipogenesis are concomitant or interdependent, and adipocytes as well as APs regulate angiogenesis by cell–cell contact and the production of cytokines^11,15,18–20^. Hence, vascularisation is a crucial parameter to take into account in generating a functional model as close as possible to the native one.

Generating vascularized AT transplants also offers an additional advantage for engraftment purposes. With lack of vascularization, grafts of tissues produced *ex vivo* often lead to short-term integration and morbidity of the donor site, along with hypoxia and cell death^21,22^. In contrast, engineered pre-vascularized tissues accelerate the connection with the *in situ* vessels and support their survival and functionality^23^. Some previous studies aimed at engineering a vascularized AT *in vitro*. The strategy usually consisted of generating adipocytes from APs, then adding ECs onto adipocytes^6,9,24–27^. However, this method does not recapitulate AT development because ECs and adipocytes are usually obtained from diverse biological sources and sometimes different organisms, which precludes the use of these engineered tissues for autologous grafts.

To counteract these problems, we aimed to produce an adipose structure by using the stromal-vascular fraction (SVF) of AT as a source of APs and endogenous ECs. According to the results obtained from different *in vitro* conditions, we opted for a unique medium as a main condition to culture the SVF in 3-D, a medium similar to that used for the culture of ECs and perivascular cells but also for APs^15,28,29^. We then assessed the ability of APs to form small spheroid-like structures with AT characteristics and functions. To maintain the adipose vascular network, we needed to develop an adipogenic milieu preventing the loss of ECs.We considered the good self-organized vessels and unilocular adipocytes obtained in our 3-D biological structures as AT organoids. In addition, these AT organoids were engrafted efficiently into nude mice by connecting their vascularization with those of the host. Our new experimental model of human AT organoids represents an invaluable tool for *ex vivo* studies mimicking *in vivo* conditions.

## Results

### Development of culture conditions for vascular network formation and adipogenesis in 2-D

**Figure 1:**
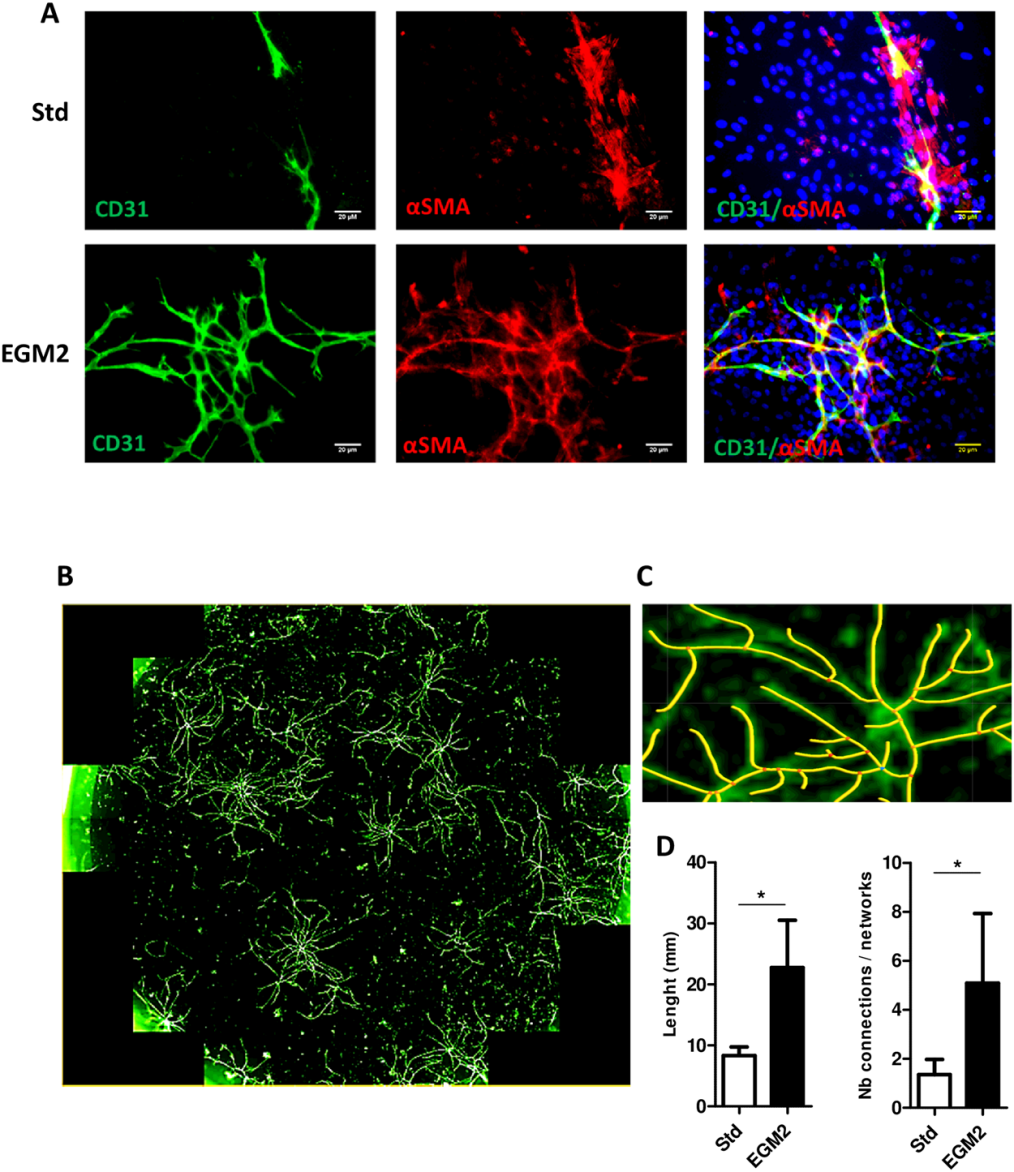
2-D capillary-like structures formed by stromal-vascular fraction (SVF)-derived cells. Adipocyte progenitors obtained from SVF were cultured under two conditions: standard (Std) or endothelial growth medium 2 (EGM2). (**A**) After several days, cultures were fixed and stained with anti-CD31 and/or anti-α-smooth muscle actin (αSMA) antibodies labelling endothelial cells (ECs) and perivascular smooth muscle cells, respectively. Nuclei were detected by DAPI staining. The preparations were then observed by confocal microscopy (bar = 20 µm). (**B**) Endothelial networks visualized by CD31 staining; the whole surface of a well was scanned by using the Operetta^TM^ screening system. (**C**) The size of endothelial capillaries and number of connections were measured. (**D**) Data are mean±SEM. *p < 0.05.

Given the importance of vessels in AT, we aimed to develop culture conditions allowing to preserve endogenous ECs along with APs. We adjusted these experimental conditions in 2-D before testing them in 3-D. Thus, after seeding human SVF in specific medium that allowed for expanding both APs and ECs as demonstrated previously by Min *et al*. (EGM2^15^), we analyzed endothelial networks and compared them with standard culture (αMEM with 10% FCS) currently used as reference for mesenchymal stem cells cultures^30^. As demonstrated by CD31 staining, vascular networks were longer and more highly branched with EGM2 than standard culture (Fig. 1). In addition, endothelial networks derived from EGM2 were lined by perivascular vascular smooth muscle cells strongly expressing the αSMA isoform (Fig. 1A). Some endothelial organizations resembled tubes (Supplementary Fig. S1) that were never observed under standard culture conditions. Therefore, EGM2 offered better conditions for obtaining ECs, which could self-organize into capillary-like structures.

We then assessed the adipocyte differentiation ability of APs present in these cultures by replacing the EGM2 medium by αMEM-2% FCS supplemented with different adipogenic molecules. After testing several combinations, the standard cocktail (mainly consisting peroxisome proliferator-activated receptor γ2 [PPARγ2] agonist rosiglitazone, 3-isobutyl-1-methylxanthine [IBMX], insulin, dexamethasone and indomethacin) was the most efficient to induce the differentiation of APs into mature adipocytes, as demonstrated by the presence of numerous lipid droplets (Supplementary Fig. S2) and by the expression of adipocyte markers (PPARγ2, lipoprotein lipase [LPL] and adipocyte lipid-binding protein [AP2], Supplementary Fig. 2). However, this standard cocktail condition was strongly detrimental for ECs because the endothelial networks disappeared (Supplementary Fig. S2). Therefore, we developed a more physiological adipogenic cocktail compatible with ECs. Besides apotransferin and insulin, which are present in classical differentiation medium, we added BMP7, a cytokine that promotes brown/beige adipogenesis *in vivo* and *in vitro*^31,32^. In addition to these molecules, we added free fatty acids (hereafter named intralipids), which can elicit both *de novo* synthesis and internalisation of fatty acids within adipocytes, thus mimicking the physiological situation. This new adipogenic cocktail led to the preservation of ECs and the formation of lipid droplets as well as increased expression of adipocyte markers (Supplementary Fig. S2), although to a lesser extent than with standard cocktail.

### Generation and characterization of vascularized adipose spheroids

**Figure 2:**
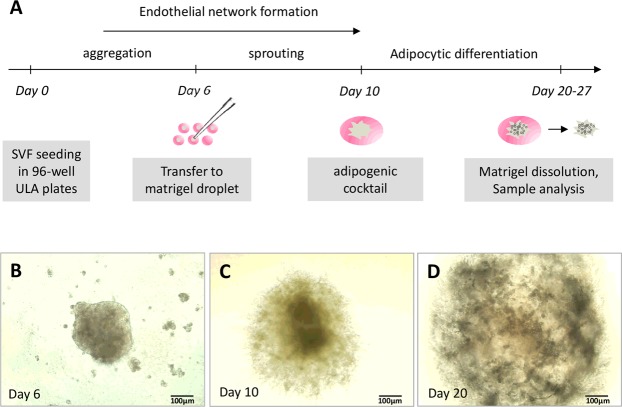
Process to obtain vascularized spheroids. (**A**) Protocol for obtaining spheroids during the 4 weeks of experiments. The formation of spheroids was followed under a photonic microscope: (**B**) spheroid in stirring liquid culture; (**C**) spheroid sprouting within Matrigel before differentiation induction; and (**D**) spheroid after 10 days of culture in adipogenic medium. (**B**–**D** are representative images).

We then asked whether features observed in 2D culture conditions could be preserved or improved in spheroids. Cells from SVF were cultured in EGM2 in ULA plastic (avoiding their adhesion on the plastic culture flasks) and with stirring (Fig. 2A). After 5 days of culture, small and dispersed cellular aggregates formed in the wells. Pipetting the medium helped with the formation of a unique spheroid per well (Fig. 2B). Six days after seeding, spheroids were incorporated into matrigel droplets and cultured further for 4 days under stirring, which allowed for sprouting (Fig. 2C). Although stirring contributed to improving the diffusion of nutrients and oxygen within the spheroid, the matrigel step was necessary to prevent spheroid condensation and the formation of a necrotic core (Fig. 2D).

**Figure 3:**
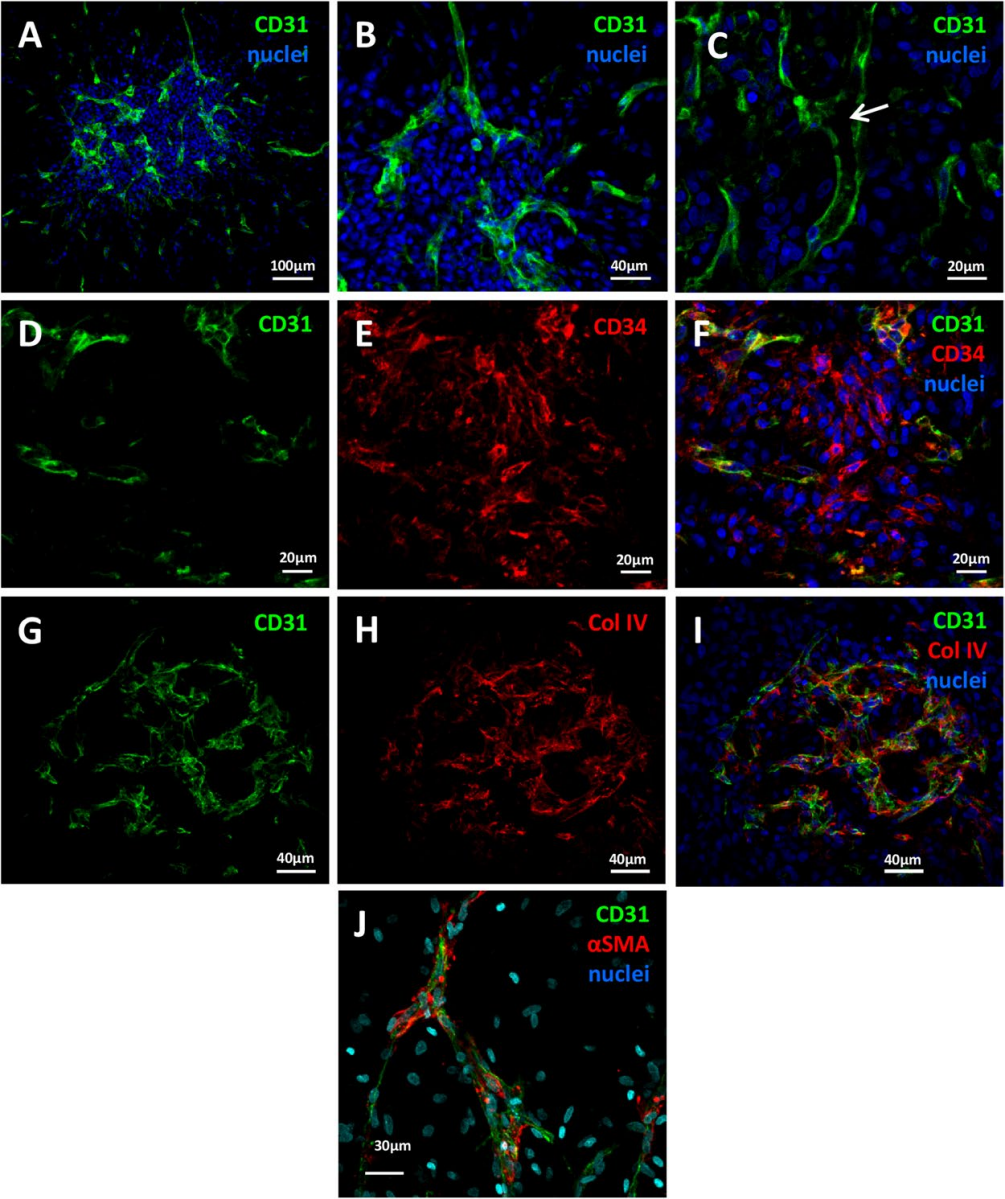
Vascularized spheroids in 3-D culture. Spheroids embedded in Matrigel were fixed and permeabilized for confocal microscopy investigation. Vessels containing ECs and perivascular cells were depicted by anti-CD31 and anti-αSMA staining, respectively (**A**–**C** and **J**). APs were detected as CD34+/CD31− cells (**D**–**F**). The basal membrane was viewed by anti-collagen IV (Col IV) staining (**G**–**I**).

At this step of the culture process, we checked the identity and localization of the different types of cells constituting spheroids, particularly ECs. CD31-expressing ECs were able to self-assemble into a dense and highly organized vascular network spanning the spheroid (Fig. 3A,B). Remarkably, tubular structures containing lumen were formed by ECs (Fig. 3C) and closely resembled the morphology of blood vessels found *in vivo*. Moreover, αSMA-positive perivascular cells (Fig. 3J) and expression of the extracellular matrix protein collagen IV of basal lamina were found along the vessels, thus completing their structural organization (Fig. 3G–I).

To test whether our culture conditions maintained the phenotype of native APs, we checked the expression of CD34, a protein known to typify APs *in vivo* but whose expression is greatly decreased in 2-D culture^33^. To distinguish APs from ECs, which also express CD34, spheroids were co-labeled with CD31. Although some cells did not have CD34 staining, a fraction of cells negative for CD31 still expressed CD34 after 4 days in matrigel (Fig. 3D–F), which indicates a preserved population of APs inside the spheroids. Therefore, spheroids generated by SVF under our 3-D conditions were well vascularized with fully formed vessels and still contained phenotypically defined APs.

**Figure 4:**
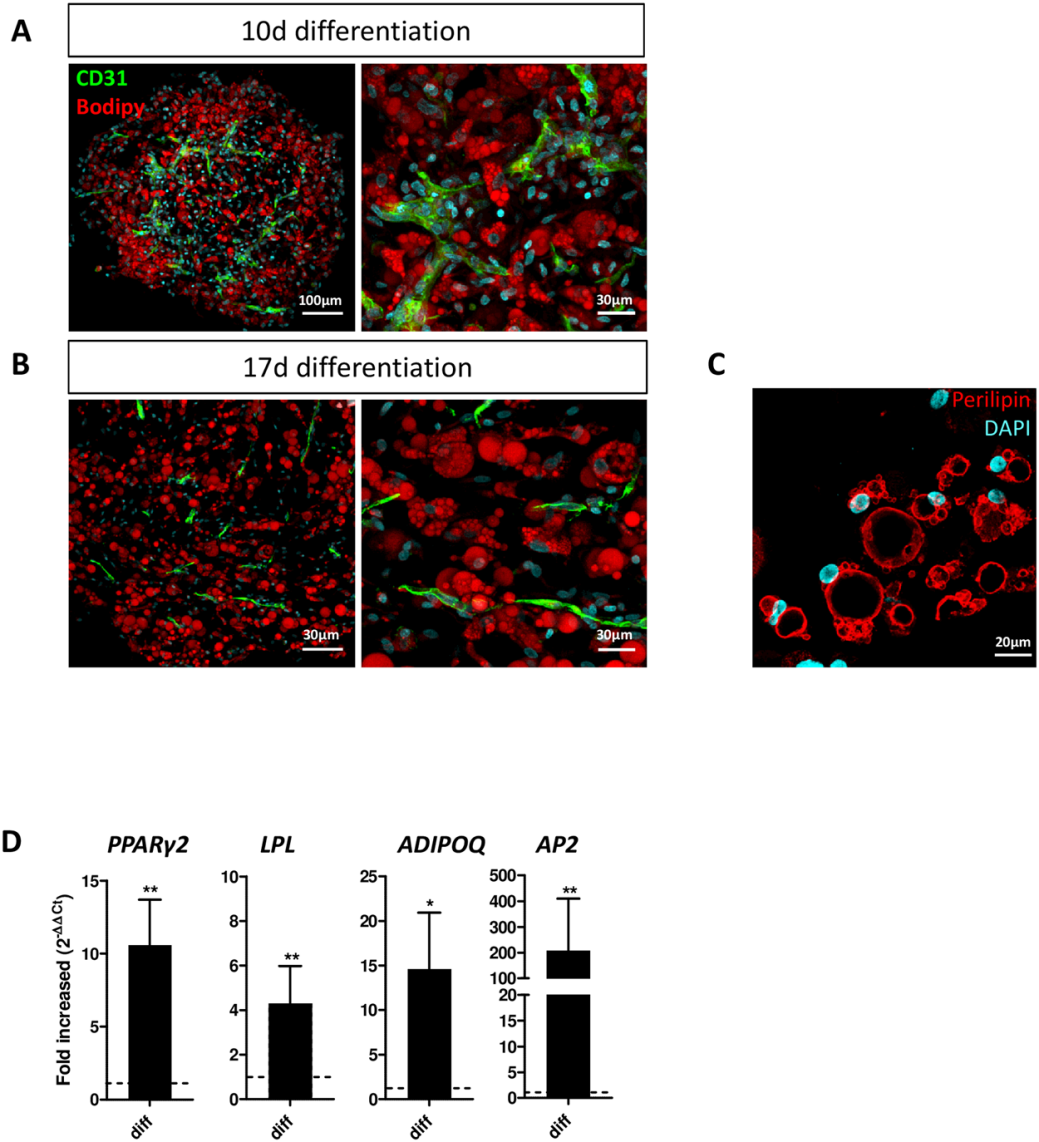
Adipocytes in vascularized spheroids. Vascularized spheroids were committed to the adipocyte lineage at up to 10–17 days. (**A**,**B**) Samples were observed by confocal microscopy. ECs were labelled with anti-CD31 antibody (**A**,**B**), and adipocytes with BODIPY (**A**,**B**) or perilipin (**C**); nuclei were stained with DAPI. The expression of adipocyte markers *PPARγ2*, *LPL*, *ADIPOQ* and *AP2* was evaluated by qRT-PCR at 10 days after the induction of spheroid differentiation versus undifferentiation (**D**). Data are mean±SEM. **p < 0.01, *p < 0.05.

Vascularized spheroids were then incubated with the αMEM-2% FCS intralipid/BMP7-based cocktail tested above under 2-D conditions and selected for its ability to preserve ECs. After 10 days of differentiation induction, some cells with large and unilocular lipid droplets were stained by the BODIPY probe (Fig. 4A–C). CD31 staining revealed that the endothelial network was intact 10 and 17 days after differentiation (Fig. 4A,B). Lipid accumulation further increased with time, many adipocytes being unilocular even at day 17 post-differentiation (Fig. 4B). This feature is reminiscent of adipocytes found *in vivo* but rarely observed in 2-D culture. Perilipin, a protein that coats the surface of lipid droplets in adipocytes, was strongly expressed and located around large vacuoles, which confirms a consistent adipocyte phenotype (Fig. 4C).

To confirm the initiation of an adipocyte-differentiation program, we analysed the expression of early and late markers of adipocyte differentiation by quantitative RT-PCR. As expected, the expression of *PPARγ2*, *AP2*, *LPL* and adiponectin Q (*ADIPOQ*) was increased after 10 days of induction with the adipogenic cocktail (Fig. 4D). Therefore, our 3-D culture conditions allowed for the formation of spheres resembling AT constituted by vessels and adipocytes.

We then assessed whether adipocytes present in spheroids maintained their plasticity toward the brown/beige phenotype after cultivating them in presence of the growth factor BMP7, previously shown to favor brown/beige differentiation^31,32^ and after an acute treatment with forskolin, a well-known browning inducer through cAMP signaling^34^. We found that UCP1 and peroxisome proliferator-activated receptor c coactivator 1 (PGC1α) mRNA levels were strongly increased following forskolin treatment, suggesting that adipocytes in spheroids preserved their plasticity toward the beige lineage (Supplementary Fig. S2). Therefore, our 3-D culture conditions allowed for the formation of spheres resembling AT containing vessels and adipocytes which maintained their plasticity.

### Transplantation of vascularized adipose spheroids *in vivo*

**Figure 5:**
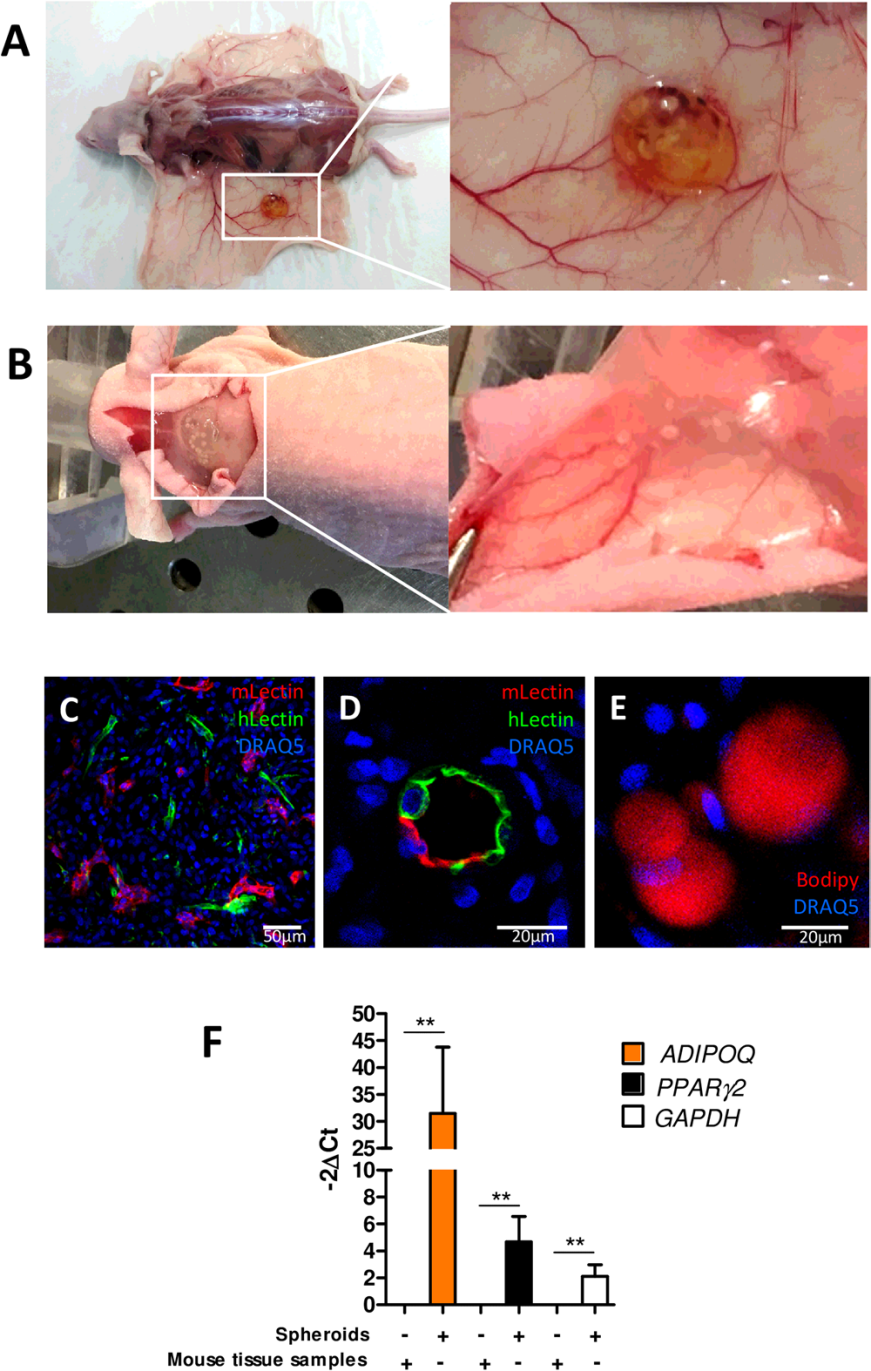
*In vivo* engraftment of vascularized adipocyte spheroids. After adipocyte differentiation, vascularized spheroids were subcutaneously injected near inguinal AT or interscapular brown AT in mice. (**A**) After day 7, transplants were harvested and sites of injections were highly vascularized. (**B**) To reveal the connection of the endothelial network of spheroids and host vascularization, both murin and human fluorescent lectins were injected in living mice. Both murin and human vessels were observed inside the adipose spheroids (**C**) as were chimeric vessels (**D**). Intra-spheroid adipocytes were stained with BODIPY (**E**). Human adipocytes remaining in spheroids were revealed by assessing the expression of the human adipocyte-specific markers *ADIPOQ* and *PAPRγ2* by qRT-PCR (**F**). Mouse tissue samples were tested to highlight the human specificity of the primers used. Data are mean±SEM. **p < 0.01.

To demonstrate that the endothelial networks formed in the differentiated spheroids were functional, we tested the potential of vascularized adipose spheroids for engrafting. After their adipogenic differentiation, 25 to 30 spheroids were implanted subcutaneously into nude mice near the subcutaneous inguinal fat pad or along interscapular brown AT. Injected mice did not show any sign of inflammation, and spheroids remained in the injection area for at least 7 days (Fig. 5A,B). The interscapular injection site was more efficient for maintaining spheroids than the inguinal AT site (not shown). After dissection, we found dense vascularisation around the plug as compared with the non-injected side (Fig. 5B). To determine whether the human vascular networks of the engrafted spheroids were connected to the recipient circulatory system, we injected both human- and mouse-specific lectins in the retro-orbital sinus before mice were euthanized. Confocal imaging revealed that the spheroids were perfused by a functional microvascular network because both lectins were easily detected (Fig. 5C). Of note, some vessels contained both human and mouse ECs, thereby suggesting chimeric vessels (Fig. 5D).

Inside the transplanted spheroids, adipocytes were still detected (Fig. 5E). Quantitative RT-PCR of the human adipocyte-specific markers *ADIPOQ* and *PPARγ2* revealed adipocytes of human origin inside vascularized adipose spheroids (Fig. 5F). These data show the functionality of vascularization of spheroids, which can easily spread and establish connections *in vivo* with the host, and reflects the good tolerance of injected cells by host. Moreover, large human unilocular adipocytes were detectable in the explants, so adipocytes-containing spheroids can be maintained *in vivo* after transplantation.

## Discussion

We developed a protocol to generate human 3-D structures *in vitro* that closely resemble the cellular architecture of human AT. These adipose spheroids could be engrafted in immune-deficient mice by connecting their own vascularization to the host one, which allowed for maintaining human adipocyte integrity.

Organoids can be generated from pluripotent or adult stem cells as well as some tissue progenitors^35,36^. Takebe *et al*. obtained organoids of different organs after their dissociation and *in vitro* reorganization^23^. However, organoids must be vascularized. Thus, the investigators performed experiments allowing for the formation of a functional endothelial network interspersed within organoids, but they needed to add ECs originating from unrelated tissue. The vascularization of AT is of primary importance as well. Indeed, there is a tight relation between ECs and APs. Abundant literature describes the role of APs in EC survival, proliferation, and organization into vascular cords *in vitro* via the secretion of bioactive molecules and cell–cell interactions^37^. The cooperation between APs and ECs in vascular assembly has also been demonstrated *in vivo* in transplantation studies^38^. In mice, adipocyte lineage tracing studies indicate that the vasculature precedes adipocyte formation and provides a niche supporting both white and brown APs^11,39^. Therefore, preserving ECs within 3-D adipose structures in culture is required for the future development of this technology. The strong interdependence between vascular progenitors and APs also underlines the crucial use of EGM2 type medium for expanding them compared to standard culture conditions. This explains why we adopt this, as done by Min *et al*.^15^ who also compared these two culture conditions and found that EGM2-like medium was necessary for vascular development and APs. In addition, they pinpointed the role of single factors VEGF, hFGF2, hEGF and IGF1 in this phenomenon. These specific conditions and molecular compositions could be gathered in more well-defined serum free medium which can thus be suitable for a clinical use^25^.

Although human adipose spheroids have been generated^40,41^, some lacked vascularization. Some research groups reported exogenous ECs (such as human umbilical vein ECs) added on adipose-derived spheroids^9,26^. Other work attempted to obtain 3-D-cultured adipose tissue models. These protocols used murine cells or organotypic-like cultures (i.e., culture of small AT explants), achieving AT-like structures with or without vascularization^27,40–44^. However, the use of exogenous ECs to form vessels inside the spheres could generate some artefacts. Indeed, the whole population of ECs is highly heterogeneous. This heterogeneity depends on the types of vessels or the tissue origin: ECs from large vessels are phenotypically and functionally different than those from smaller vessels such as sinusoid, microvessels and arterioles. Moreover, ECs from liver, the central nervous system, and bone marrow have common features but also consistent differences depending on the function of the tissue where they reside^45^. To improve the development of spheroids, we established culture conditions allowing for spheroid vascularization with endogenous ECs (i.e. of AT origin). By keeping endogenous ECs surrounded by perivascular cells and APs, our vascularized AT model could recreate the native environment, which would explain why our AT-organoids were relatively well maintained even *in vivo*. Besides being important for adipocyte biology, this structure could also allow for study of the different actors of the perivascular niche that play a significant role in the fate of APs^20^.

To obtain such results, we needed to set up culture conditions that enabled both the maintenance of vascular integrity and the differentiation of adipocytes. Contrary to pharmacological molecules commonly used to initiate adipogenesis *in vitro*, our more physiological adipogenic cocktail preserved ECs. The pharmacological factors used (IBMX, rosiglitazone and indomethacine) might have issues related to growth and/or viability of ECs^46,47^, which led to inhibition of spheroid vascularization, as reported by others^46–48^. Although pharmacological factors did not have any toxic effect on ECs in monocultures (HUVEC, data not shown), the differential responses of ECs to these molecules might be explained by the type of cultures (mono versus co-cultures), highlighting the possible role of APs in ECs survival.

Despite reduced adipogenic induction, we were still able to generate unilocular adipocytes, a mechanism tightly related to physiological situations. Indeed, fatty acids (included in our differentiation medium) play a major role in the adipogenesis process, both by internalization in lipid droplets and activation of PPARγ, the master regulator of adipogenesis^16^. This inclusion strengthens the similarity of adipose organoids and native AT and constitutes an interesting model to further study the link between adipogenesis and angiogenesis in humans.

The vascularization of adipose organoids is important for the reasons described above but also for the crucial functions that ECs play in the normal and pathological context of AT even if unclear. Modulation of the adipose vasculature has emerged as a potential strategy to treat obesity. Intriguingly, both repression and induction of AT vasculature can improve metabolism in obesity, and the effect of anti-angiogenic treatments varies depending on the patient’s age and obesity status^49^. Nevertheless, these studies underline the importance of the EC source. Therefore, ECs from patients’ AT would better help predict the outcome in drug discovery.

Even after their xeno-transplantation in mice, human ECs and adipocytes were still detected in our study. Also, chimeric vessels were obtained. This observation underlines that spheroid vascularization is viable and functional, with strong ability to be integrated with the host vasculature. Of note, addition of matrigel during spheroid implantation helped their vacularization as described by Laib *et al*.^50^, but other biomaterials such as fibrin should be tested for clinical perspectives. The fact that human adipocytes were found to be preserved in the host environment suggest that they could be used in numerous metabolic studies. Nevertheless, we lack *in vivo* functional investigations for evaluating the relevance of this model for the ability of white adipocytes to be converted into beige cells or to stock and transform metabolites, as is normally done for this type of cell.

Overall, our adipose organoids have several potential benefits. First, the generation of mature unilocular adipocytes together with an organized vascular network provides a more physiological platform to study human ATs *in vitro* under normal and pathological contexts. Second, because of the ability of adipose organoids to respond to browning inducers, they are a valuable model to decipher the mechanisms involved and to test new drugs for therapy of obesity and associated disorders. Third, all of these studies could be performed in different settings such as for large-scale drug screening of micro-chip technologies. Finally, they constitute an interesting model to further study the link between adipogenesis and angiogenesis in humans. These organoids could also answer the need for vascularized autologous AT for transplantation, because we found that they could be transplanted in mice and anastomosed promptly with the host vasculature.

In conclusion, we herein developed a model of vascularized AT-like spheroids using SVF of AT as a source of APs and ECs. Differentiated spheroids transplanted into mice connected to the recipient circulatory system, highlighting the functionality of the endothelial network and the relevance of adipospheres for multiple applications.

## Methods

### SVF isolation from human adipose tissue

Human SVF was isolated from subcutaneous adipose tissue obtained from human non-obese donors (body mass index <26 kg/m^2^) undergoing elective abdominal dermolipectomy (plastic surgery department, CHU Toulouse, France). Our experimental protocols were approved by French research ministry’s institutional ethics committee (No: DC-2015-23-49) and informed consent was obtained from all subjects in line with current regulations (no subjects under 18 were included). The AT was dissociated and underwent enzymatic digestion in αMEM (Life Technologies, Carlsbad, CA, USA) supplemented with penicillin and streptomycin (p/s) without serum, with 13.6 U/mL collagenase NB4 (Serva Electrophoresis; Heidelberg, Germany) and 10 U/mL dispase (Roche; Bâle, Switzerland) for 45 min at 37 °C under stirring. The digestion was filtered with a 100-µm Nylon net filter (Steriflip, Millipore, Burlington, MA, USA) and centrifuged (600 g, 10 min). The pellet containing SVF was resuspended in αMEM with p/s without serum, and erythrocytes were lysed for 3 min at 4 °C by using lysis buffer. Lysis was stopped with the addition of αMEM with p/s without serum. To eliminate the lysed cells, another centrifugation was performed (600 g, 10 min). The SVF obtained was then resuspended either in endothelial growth medium 2 (EGM2; PromoCell; Heidelberg, Germany) or αMEM complemented by 10% Fetal Calf Serum (FCS, standard culture medium, Life Technologies), and 1 U/µL pulmozyme (Life Technologies) was added. Cells were counted by using an automatic cell counter (Countess, Invitrogen; Carlsbad, CA, USA) and seeded in EGM2 or standard medium at 4000 cells/cm^2^ for 2-D cultures or 25 000 cells/well in 96-well ultra-low attachment (ULA) plates with a round bottom (Costar, ThermoFisher; Waltham, MA, USA) for 3-D culture.

### Endothelial networks in 2-D conditions

After 2 weeks of expansion, SVF-derived cells were seeded at 1 × 10^5^ cells/cm^2^ in a flask coated with a solution of 0.1% (v/v) gelatin. At day 10, cells were fixed, permeabilized with 0.1% (v/v) triton X100 (Sigma) and incubated with the antibodies anti-CD31 (ref: JC70A, Dako; Santa Clara, CA, USA) and/or anti-α-smooth muscle actin (α-SMA; ref: 202M-9, Sigma) to evaluate the formation of vessels. 4′,6-diamidino-2-phenylindole (DAPI, Sigma) was used to stain nuclei. Cells were observed under a Nikon Eclipse TE2000-S confocal microscope (Nikon; Champigny sur Marne, France). Quantification of vessel length and the number of vessels involved the use of the FilamentTracer module of Imaris software (Bitplane; Zurich, Switzerland).

### Adipocyte differentiation in 2-D conditions

SVF was seeded at 100,000 cells/cm^2^ in EGM2 and cultured for 8 days before differentiation. Cells were then cultured for 14 days in different adipogenic media: the classical medium (αMEM supplemented with 2% FCS, 1 µM Dexamethasone, 60 µM Indométhacine, 2 µM Rosiglitazone, 5 µg/mL Insulin, and 450 µM (3-isobutyl-1-methylxanthine, IBMX, for the first 3 days), or αMEM-2% FCS supplemented with Intralipids (1/100), Insuline (5 µg/mL), or Bone Morphogenetic Protein 7 (BMP7, 50 ng/mL)^32^.

### Spheroid formation, incorporation in matrigel and differentiation

For each culture step, the medium was changed every 2–3 days, pipetted for cell aggregation and incubated at 37 °C under stirring (70 rpm). When cell aggregates formed a unique spheroid per well, usually six days after the SVF was seeded, spheroids were incorporated in 40 µL matrigel (growth factor-reduced, Corning; New York, USA), one by one, and seeded in EGM2 in ULA and ultra-low cluster 24-well plates with a flat bottom (Corning). This step was essential to avoid the formation of a necrotic core since matrigel inclusion allows cell migration, generating a spheroid with lower cell density and better access to nutrients and oxygen. After 4 days, spheroids were differentiated by using an adipogenic cocktail composed of intralipids 0.2% v/v (Sigma I141; St. Louis, MO, USA), 50 ng/mL BMP7 (R&D Systems; Bio-Techne, Minneapolis, MN, USA), insulin 5 µg/mL and apotransferin 10 µg/mL diluted in αMEM and p/s with 2% FCS (Life Technologies). Spheroids could be then maintained in culture for at least 17 days after differentiation without any sign of cell death. Thermogenic genes were induced in spheroids by treating them with 50 μM forskolin (Tocris; Bio-Techne) for 4 h.

### RNA extraction, reverse transcription and quantitative PCR

After the Matrigel was washed in cold phosphate buffered saline (PBS), spheroids were lysed in Qiazol, and total RNA was extracted by using the Qiagen RNeasy Micro kit (Qiagen; Hilden, Germany). Reverse transcription involved 219–1000 ng RNA with the High Capacity cDNA Reverse Transcription kit (Applied Biosystems; Foster City, CA, USA). cDNA was synthetized in a thermal cycler (2720 Applied Biosystems) with the program 10 min at 25 °C, 120 min at 37 °C and 5 min at 85 °C.

qPCR involved the SYBR Green PCR Master Mix (Applied Biosystems) with 375 nmol/L of the primers listed in Table 1. After centrifugation, reactions involved use of the StepOne device (Applied Biosystem). Data were analysed by using StepOne software. Relative expression was calculated by the ΔΔCT method and normalized to the reference gene PPIA.

**Table 1:** Sequences of primers used in QRT-PCR.

Target	Forward primer	Reverse primer
*PPIA*	GCCGAGGAAAACCGTGTACTA	GCCGAGGAAAACCGTGTACTAT
*GAPDH*	TTGACAAAGTGGTCGTTG	CTGGCGCTGAGTACGTCG
*PPARγ2*	GATACACTGTCTGCAAACATATCAC	CCACGGAGCTGATCCCAA
*AP2*	AAACTGGTGGTGGAATGCGT	GCGAACTTCAGTCCAGGTCA
*LPL*	GGTCGAAGCATTGGAATCCAG	TAGGGCATCTGAGAACGAGTC
*ADIPOQ*	TGCCCCAGCAAGTGTAACC	TCAGAAACAGGCACACAACTCA
*UCP1*	GTGTGCCCAACTGTGCAATG	CCAGGATCCAAGTCGCAAGA
*PGC1α*	CCGCACGCACCGAAA	TCGTGCTGATATTCCTCGTAGCT

### Immunofluorescence staining of spheroids

Spheroids were washed with cold PBS (Life Technologies) until the disappearance of Matrigel. Spheroids and explanted plugs were fixed with formaldehyde 3.7% (Sigma Aldrich) overnight at 4 °C and washed 3 times with PBS. They were embedded in 4% agarose and cut into 150-µM sections by using a VibroSlice (Campden Instruments; Lafayette, IN, USA). Sections were permeabilized and blocked in PBS + 0.2% Triton X100 + 5% serum (horse or donkey) (Sigma) for 30 min at room temperature, then incubated overnight at 4 °C with primary antibodies diluted in PBS + 0.2% Triton X100 + 5% serum. The following antibodies were used: anti-CD31 (ref: JC70A, Dako), anti-perilipin A/B (ref: P1873, Sigma), anti-laminin (ref: M3F7, Developmental Studies Hybridoma Bank [DSHB]; University of Iowa, Iowa City, IA, USA), anti-collagen IV (ref: C4, DSHB), and anti-α-SMA (ref: 202M-9, Sigma). Secondary antibodies coupled with Alexa fluorochromes 488, 555, 594, 647 (Life Technologies) were diluted 1:400 in PBS + 0.2% Triton X100 and added to sections for 1.5 hr at room temperature. Sections were then washed 3 times and stained with the nuclear marker DRAQ5 (1:1000; BioStatus; Loughborough, UK) or DAPI 1:10000 for 15 min. To mark adipocyte lipids, sections were incubated with BODIPY 1:1000 (Invitrogen; Carlsbad, CA, USA) for 30 min before the nuclear marker and washed 3 times. Sections were observed by Operetta^TM^ screening system (PerkinElmer; Courtaboeuf, France) or by confocal microscopy (Zeiss LSM-780 or Zeiss LSM-510; Zeiss; Oberkochen; Germany). Images were processed by using Fiji (an open-source platform for biological-image analysis^51^) and Imaris software (Bitplane).

### Implantation of spheroids and mice experimentation

Female NMRI/Nu (nu/nu) mice 6 weeks old were obtained from Elevage Janvier (Le Genest Saint Isle, France). All experiments were performed with 7-week-old mice. Mice were housed in a barrier facility with high-efficiency particulate air-filtered racks. Animals were maintained according to the guidelines of the European Community Council (2010/63/UE) and experimental protocols were approved by the French ethics committee (protocol reference no.: 13/5273/B/02). In order to concentrate the spheroids in a small volume, the matrigel was dissolved in cold PBS. A mixture containing 1.2% methylcellulose (w/v), one-third 10 mg/ml fibrinogen, and one-third EC basal medium at a 1:1:1 ratio supplemented with 1 µg/ml vascular endothelial growth factor and fibroblast growth factor 2 (R&D Systems) was prepared. A total of 25–30 spheroids was resuspended in 200 µl of this mixture^50^. Just before injection in mice, 3 U thrombin and 200 µl Matrigel was added. Recipient mice were anesthetized by isoflurane inhalation and spheroids were injected into their right flank with a large diameter syringe (19G) to avoid damaging spheroids. After 1 or 2 weeks, human-specific lectin (Biotinylated Ulex Europaeus Agglutinin I) or mouse-specific lectin (Rhodamine Griffonia Simplicifolia I; CliniSciences, Nanterre, France) was injected in the retro-orbital vein of recipient mice, and 20 min later, mice were euthanized by cervical dislocation. After plug excision, spheroids showed a round morphology indicating that they were intact after transplantation. Plugs were fixed overnight at 4 °C in 4% paraformaldehyde. To visualize human vasculature, staining was performed with Alexa Fluor 488 streptavidin (Life Technologies).

### Statistical analysis

Quantitative data are expressed as mean ± SEM. Groups were compared by a one-sample *t* test and a Gaussian distribution-free Wilcoxon matched pairs test otherwise. The statistical significance was set at p < 0.05. Statistical analysis was performed with Prism 5 (Graphpad^TM^).

## Supplementary information

Supplementary information
