# Supplementary material for: Human adipose stromal-vascular fraction self-organizes to form vascularized adipose tissue in 3D cultures
Source: Sci Rep. 2019 May 10;9:7250. doi: 10.1038/s41598-019-43624-6 (PMC6510792; doi:10.1038/s41598-019-43624-6)

Human adipose stromal-vascular fraction self-organizes to form vascularized adipose tissue in 3D cultures

Sandra Muller[1], Isabelle Ader[1], Justine Creff[1,2,3], Hélène Leménager[1], Pauline Achard[1], Louis Casteilla[1], Luc Sensebé[1], Audrey Carrière[1], Frédéric Deschaseaux[1*].

Supplemental figure legends

Supp. Figure S1. SVF cells were cultured in EGM2; capillary-like structures were observed by anti-CD31 staining for endothelial cells. These capillaries seemed to form tubes with lumen, which were never detected with standard medium.

Supp. Figure S2. Several types of adipogenic media were tested on vascularized cultures: a medium containing a cocktail of molecules currently and widely used and medium containing free fatty acids (intralipids) complemented with other adipogenic molecules (insulin or BMP7). Lipid droplets and CD31-positive endothelial networks were observed by confocal microscopy (**A**: fluorescent staining; **B**: phase contrast). (**C**) The expression of several adipocyte markers (*PPARγ2*, *LPL* and *AP2*) was determined by qRT-PCR. (D) Expressions of markers of beige adipocyte, *UCP1* and *PGC1A*, were also tested by qRT-PCR before and after adipogenic differentiation (Diff) and then with or without addition of 50 µM forskolin (Fsk). Data are mean±SEM. *P<0.05.; ***p<0.001. ns: not significant.

Supplemental figure 1

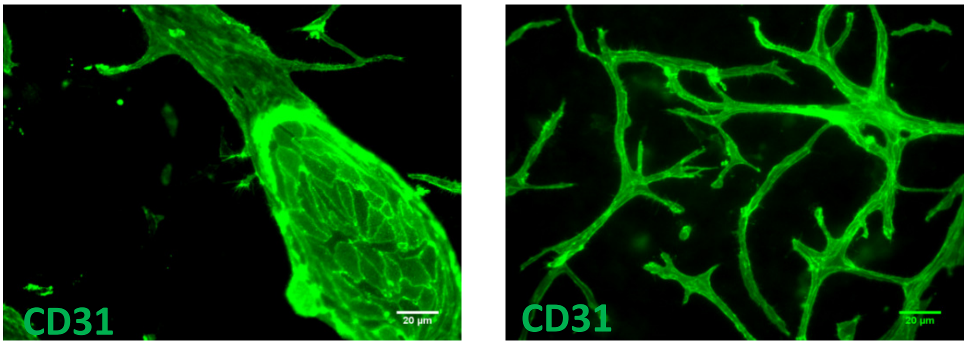

Supplemental figure2

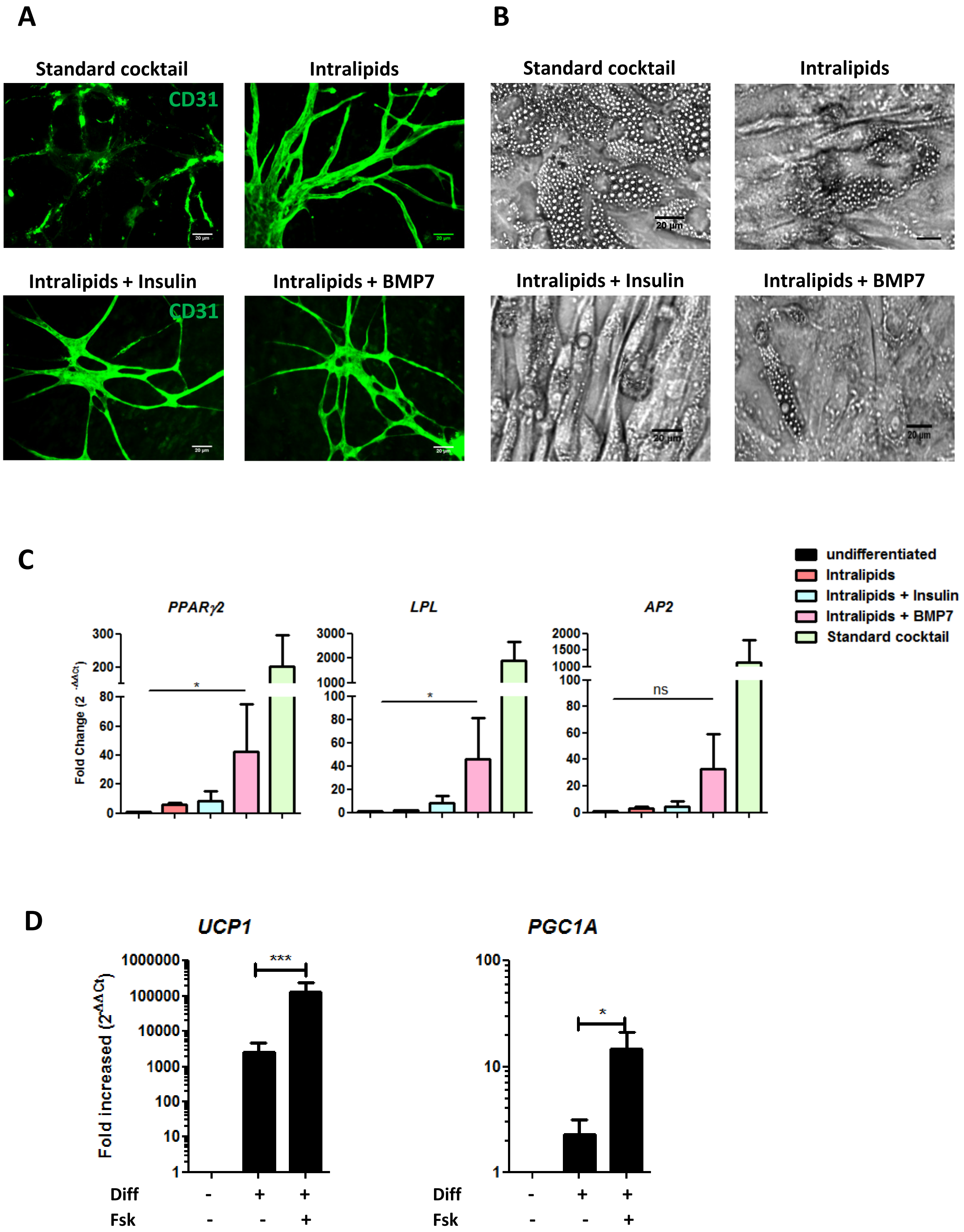

Supplement: Supplementary file 1 — Supplementary information [file 41598_2019_43624_MOESM1_ESM.pdf]
